# Equivalence of feature-tracking–derived myocardial strain across spatial resolution and compressed sensing acceleration

**DOI:** 10.1007/s10554-025-03594-5

**Published:** 2026-01-05

**Authors:** Baptiste O. P. Wyssa, Lindsey A. Crowe, Jean-François Deux, Teodora Chitiboi, Jens Wetzl, Jean-Paul Vallée, Carl G. Glessgen

**Affiliations:** 1 Department of Radiology, University Hospitals of Geneva (HUG), Geneva, Switzerland; 2 Research & Clinical Translation, Magnetic Resonance, Siemens Healthineers AG, Erlangen, Germany

**Keywords:** Heart, Magnetic resonance imaging, cine, Cardiac imaging techniques, Image processing, Computer-Assisted

## Abstract

Reference values for myocardial strain as measured by feature-tracking (FT) MRI cine images are known. As these values may be impacted by source image resolution, we aimed at assessing strain parameters across low-resolution single-shot and high-resolution segmented cine images using different FT software. Thirty-seven patients (median age 40 years [IQR: 26–52]; 13 female) with same-session segmented high-resolution (SEG) and compressed sensing-accelerated single-shot (CS) cines from a 1.5T system (Magnetom Sola, Siemens Healthineers) were retrospectively included. Commercial (CVI42, Circle) and prototype software (TrufiStrain, Siemens Healthineers), FT and DRA, respectively, were used for analysis. Peak circumferential and radial strain were calculated globally and segmentally using individual segment peaks (peak-segment) or segment strain at the global peak frame (peak-frame). Equivalence was tested with the two one-sided test (TOST) for equivalence (90% CI), and Spearman correlation (ρ) with left ventricular ejection fraction (LVEF) was calculated. A significance level of α = 0.05 was used. Strain values differed significantly between SEG and CS images for most combinations, except peak-segment global circumferential strain (GCS) using DRA (SEG: −16.0% [− 17.5, − 14.4] vs. CS: −16.8% [− 17.8, − 14.9]; *p* = 0.072), which met equivalence criteria (mean difference − 0.38%, CI90: −0.72% to − 0.05%). Using CS images, DRA-derived GCS correlated more strongly with LVEF (ρ = −0.780) than did FT-derived GCS (ρ = −0.514). In most cases, myocardial strain was not equivalent across accelerated low-resolution and high-resolution segmented images. Only one of eight tested combinations (strain parameter, software, peak definition) met statistical equivalence, showcasing the need for novel processing methods to mitigate variability.

**Graphical abstract:**

**Figure:**
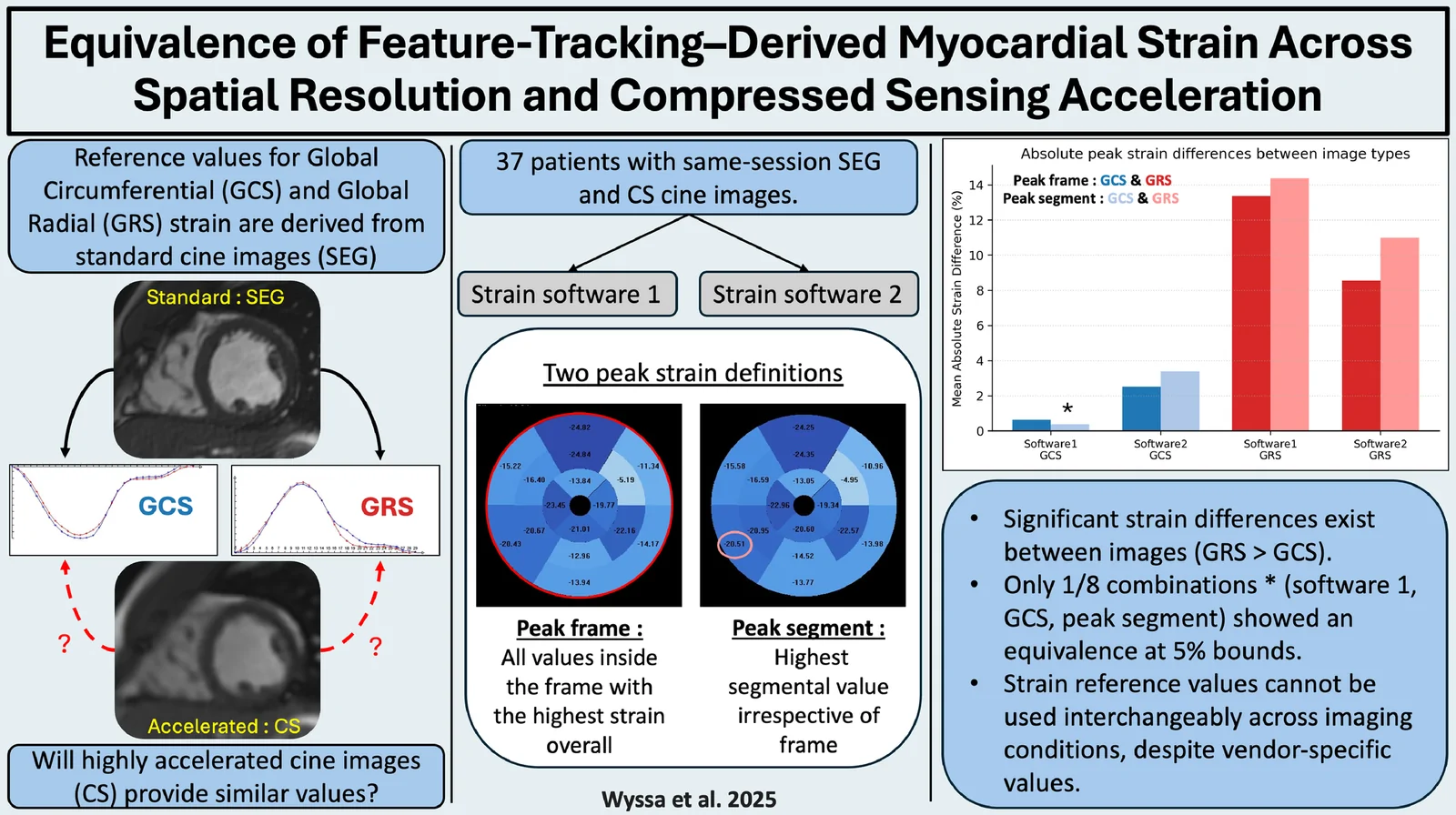

## Introduction

Cardiac strain analysis has become integral to cardiac MRI, offering diagnostic and prognostic value beyond traditional volumetric measures such as left ventricular ejection fraction (LVEF) [1–4]. Among strain quantification methods, feature-tracking (FT), which derives strain from standard cine images without additional acquisitions, has become widely adopted due to its practicality [5, 6]. While reference values have been published [7–9], these norms do not fully account for vendor variability [10], and even less so for the wide array of imaging protocols in current practice [11, 12].

Indeed, cine image quality can vary significantly due to patient characteristics (e.g., larger body habitus, impaired breath-holding capability, arrhythmias) or specific contexts requiring accelerated acquisitions, specifically real-time imaging and compressed sensing (CS) [13–15]. While these methods preserve diagnostic efficacy [16, 17], spatial and temporal resolution can be altered, potentially impacting strain measurements [18, 19]. Although prior studies have separately evaluated the impact of reduced spatial resolution [20] or combined CS-real-time acceleration on FT-derived strain [21, 22], the combined effects of both have not been investigated yet. Determining whether these two factors together further influence strain assessments is therefore important for clinical reliability, particularly given the growing adoption of accelerated protocols in routine practice.

In this study, we further explore whether software-based methodological distinctions translate into differing levels of consistency when cine imaging is accelerated and rendered in lower spatial resolution. The combined effects of reduced spatial resolution and acquisition acceleration will be assessed using two different strain-tracking software packages. Both packages rely on optical flow-based methods, but with slightly different mathematical strategies and assumptions to estimate myocardial motion. Notably, one software focuses primarily on the myocardial borders, whereas the other analyzes the entire myocardial tissue region, providing both global and layer-specific strain values, and reportedly achieving improved reproducibility [23–25].

We hypothesize that the combined effects of reduced spatial resolution and CS reconstruction will lead to differing levels of agreement across software compared to reference strain values derived from high-resolution cine imaging.

## Methods

### Study population

This retrospective, single-center study compared peak circumferential and radial strain measurements across image resolution types within the same patients, as obtained from two strain tracking software packages. The local institutional review board (IRB) approval was obtained (ID: 2024 − 01574), and the requirement for informed consent was waived due to the retrospective nature of the study and the use of pseudonymized imaging data. All cardiac MRI (CMR) cases with same-session conventional and compressed sensing cine imaging data were retrospectively identified from our hospital’s picture archiving and communication system (acquisitions: 07/2023-06/2024). The dual acquisitions were performed as part of internal quality control, independent of patient profiles. Cine sequences were assessed visually by an experienced cardiac radiologist (CG, 4 years’ experience, level 3 certification), and cases with incomplete ventricular coverage, significant artifacts due to arrhythmia or respiratory motion in either acquisition, or those with metallic devices were excluded.

### Magnetic resonance cine data acquisition

All CMR scans were performed on a 1.5T clinical system (MAGNETOM Sola, Siemens Healthineers AG, Forchheim, Germany) using an 18-channel body coil in the supine position. Each examination included both conventional segmented (SEG) and compressed sensing real-time (CS) cine acquisitions without contrast material. Details on the CS reconstruction scheme have been described previously [17]. The SEG cine images were acquired in breath-hold with multiple contiguous short-axis slices covering the left ventricle, using retrospective electrocardiographic (ECG) gating. CS cine images were ECG-triggered, single-shot acquisitions in end-expiratory breath-hold, covering the same short-axis planes. Long-axis views (2- and 4-chamber) were acquired for SEG but were not available for CS. Each cine sequence yielded 30 frames per cardiac cycle, with 30 frames calculated from the retrospectively triggered SEG, and similarly, interpolation to 30 frames from an acquisition per slice slightly longer than 1 RR for CS. Acquisition parameters of both SEG and CS are detailed in Table 1.

**Table 1:** Imaging parameters across sequences

Parameter	Standard cine (SEG)	Accelerated cine (CS)	*P*-value
ECG gating	Retrospective	Prospective	–
TE/TR (ms)	1.2/2.7	1.05/2.5	–
FOV (mm, % of phase dimension)	360 × 288 (80%)	500 × 250 (50%)	–
Image matrix	224 × 192, INTP: 448 × 384	208 × 130	–
Source spatial resolution (mm)	1.6 × 1.6, INTP: 0.8 × 0.8	2.4 × 2.4	–
Temporal resolution (ms)	35.4 [35.2–35.6]	34.4 [32.0–36.9]	0.149
Segments	13	12	–
Slice thickness (mm)	8	8	–
Flip angle (degree)	64 (variable)	55 (fixed)	–
Bandwidth (Hz/pixel)	930	962	–
Cardiac phases (n)	30	27 [24–30], INTP: 30	–
Slices per breath-hold (n)	2	14–16	–
Acceleration Factor (n, mode)	3 (GRAPPA)	9 (CS)	–
Acquisition duration (SAX)	3:45 min	0:20 min	–

Values are presented as median values with inter-quartile range [25–75] and compared using the Wilcoxon signed-rank test when appropriate. A p-value of 0.05 was considered statistically significant. ECG = electrocardiogram, TE/TR = echo time/repetition time, FOV = field of view, INTP = interpolated, SAX = short-axis block

### Image processing and strain analysis

Image analysis was performed using two software packages: the commercially available CVI 42 (version 6.1.1, Circle Cardiovascular Imaging Inc., Calgary, Canada) and the prototype software TrufiStrain (version 3.0, Siemens Healthineers AG, Forchheim, Germany). For clarity, the two will be respectively referred to as FT (feature-tracking) and DRA (deformation registration analysis).

For quality control, automated contours from both software packages were reviewed by a single cardiac radiologist (CG) following a standardized procedure. In accordance with established recommendations, basal slices displaying an incomplete myocardial ring or outflow tract and borderline-quality apical slices were systematically excluded [11]. The same slices were consistently excluded across the SEG and CS datasets to ensure identical myocardial coverage. For each patient, identical slices originating from the SEG and CS datasets were selected and processed within each software package. The FT software initially performed automated detection of endocardial and epicardial borders. Cine images were similarly analyzed with DRA, first using deep-learning-based myocardial segmentation to define the region of interest, then inverse-consistent deformable image registration to calculate deformation fields and strain values, as described previously [26, 27]. Automated contouring results from both software packages were visually verified by the same radiologist, blinded to all strain outputs, who applied identical manual corrections where required.

**Fig. 1:**
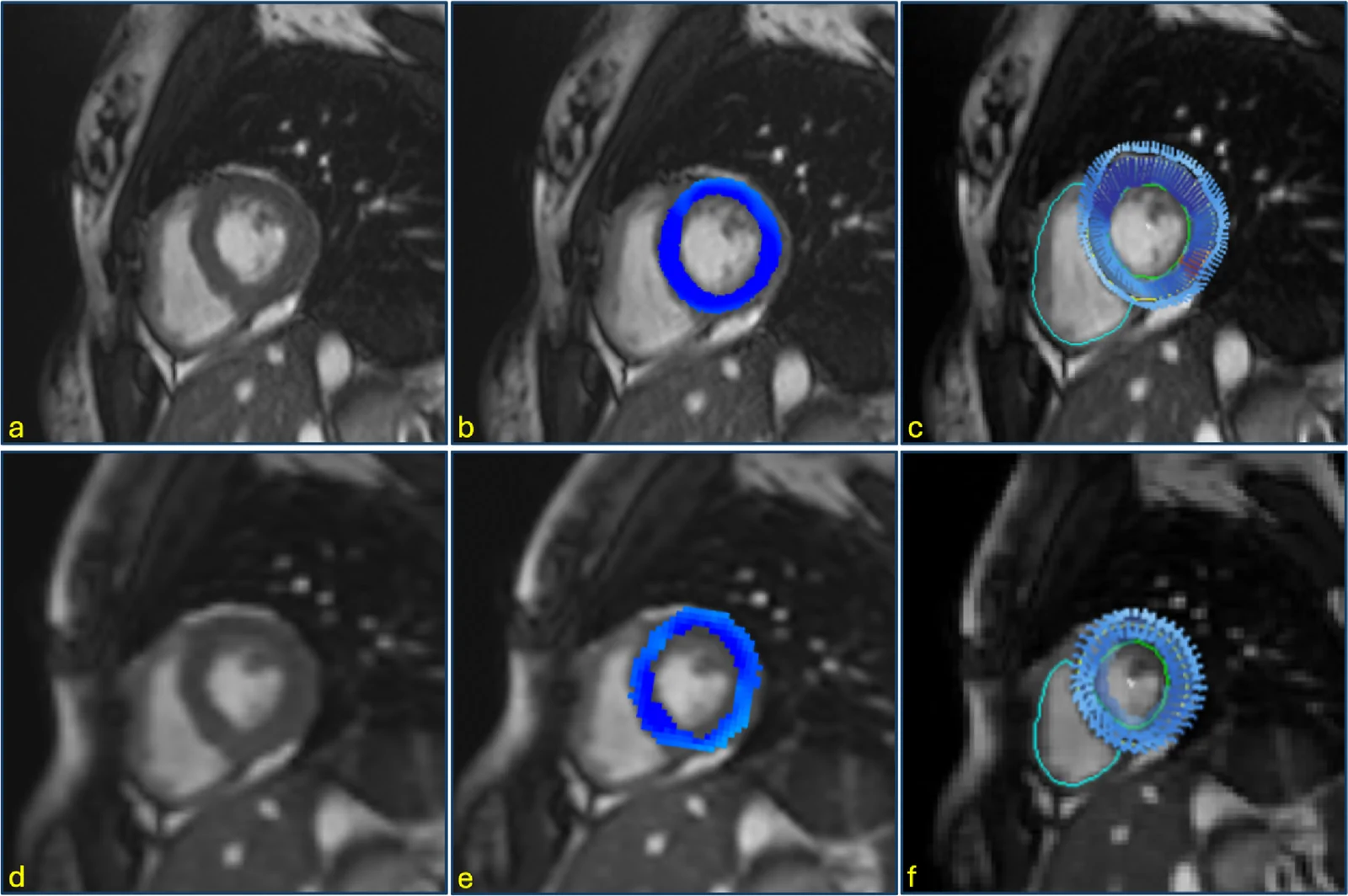
Short-axis mid-ventricular cine frames from the same patient acquired in high- (**a**) and low- low-resolution (**b**) and rendered in systole. Notice the sharpness difference of papillary muscles, lung, and liver vessels between both resolutions. Deformation fields of circumferential strain obtained from commercial (**b**, **e**) and prototype (**c**, **f**) software. The difference in resolution is also visible from the pixelated aspect of the deformation fields (**e**, **f**)

Circumferential and radial strain measurements were obtained from short-axis slices according to the standard American Heart Association 16-segment model. Strain values were obtained for each cardiac segment, each cardiac frame (30 frames per cardiac cycle), each image type (SEG and CS), and both software packages (FT, DRA). Exemplary strain outputs by sequence and software are shown in Fig. 1. Left ventricular ejection fraction (EF) was assessed on SEG images using the commercially available FT software, as per standard clinical use in our institution.

In this study, only peak strain values are reported, at either the segmental or the global level (mean of 16 AHA segments). To ensure clarity, circumferential and radial strain values are denoted as global circumferential strain (GCS), global radial strain (GRS), segmental circumferential strain (SCS), and segmental radial strain (SRS). Two distinct methods were used to define peak strain and choose the corresponding value: peak-segment and peak-frame. In the peak-segment approach, peak strain was defined as the most extreme value observed within each segment, and global values (GCS, GRS) were computed as the mean of these segmental peaks, regardless of their temporal occurrence. In contrast, the peak-frame approach identified the time frame in which the mean strain across all segments reached its maximum absolute magnitude, using these segmental values for both global and segmental analysis.

### Statistical analysis

Because data were non-normally distributed (as per Shapiro-Wilk testing), median and interquartile range (IQR) are reported, and paired comparisons were conducted using the Wilcoxon signed-rank test. Equivalence between SEG and CS was evaluated using a two-one-sided test (TOST) procedure [28], with a 5% relative margin (derived from mean strain across SEG and CS). A post hoc power analysis was performed to confirm the robustness of the findings. Agreement between SEG and CS acquisitions was further assessed by Bland–Altman analysis, with mean bias and limits of agreement (± 1.96 standard deviations) being reported.

The relationship of GCS and GRS with ejection fraction (EF) was assessed using Spearman’s rank correlation coefficient (ρ). In addition, the impact of the native number of SEG and CS cardiac frames (before interpolation) on the magnitude of GCS and GRS differences (Δ = ∣CS − SEG ∣) was assessed using Spearman’s rank correlation coefficient (ρ).

A significance level of α = 0.05 was applied. All analyses were performed in Python (version 3.9.6, Python Software Foundation) using pandas for data manipulation, SciPy for statistical testing, and matplotlib for visualization.

## Results

### Study population

A total of 50 CMR cases with same-session conventional (SEG) and compressed sensing (CS) cine acquisitions were identified. Cases with incomplete ventricular coverage (*n* = 1), significant artifacts due to arrhythmia (*n* = 1), respiratory motion (*n* = 7), or metallic devices (*n* = 2) were excluded. In addition, two cases presented a misregistration of their trigger times due to failed cycle capture during acquisition, leading to a failed analysis by DRA, and were excluded. Thirty-seven cases were included in the final analysis. The clinical characteristics of the study population, including indications for CMR, are summarized in Table 2. There was no significant difference in native temporal resolution, expressing the native duration of a cardiac cine frame before interpolation, between SEG (35.4 ms [35.2–35.6]) and CS (34.4 ms [32.0–36.9]) datasets (*p* = 0.149).

**Table 2:** Characteristics of the study population

Characteristics	Patients (*n* = 37)
Age (y)	40 [26–52]
Sex (female/male)	13/24
Height (cm)	172 [166–176]
Weight (kg)	74 [56–85]
BMI (kg/m2)	24 [20.6–27.8]
Heart rate (bpm)	65.1 [58.6–71.0]
Left ventricular ejection fraction (%)	56.0 [53.7–60.6]
Clinical Indication (n)	
Aortic disease	4
Congenital vascular disease	5
Coronary artery disease	9
Heart failure	2
Hypertrophic cardiomyopathy	2
Myocardial fibrosis assessment	4
Myocardial infiltration	2
Myocarditis	5
Pericardial disease	1
Viability imaging	3

Values are presented as median values with inter-quartile range [25–75], or as counts

### Strain comparison between sequences

GCS, GRS, SCS, and SRS values obtained by both software were compared between SEG and CS sequences, Table 3. Except for one, all comparisons showed significant differences between acquisitions, with CS images consistently yielding significantly lower absolute circumferential and radial strain compared to SEG. GCS measured by DRA did not differ significantly between acquisitions, when using the peak-segment approach (SEG: −16.0% [−17.5%: −14.4%] vs. CS: −16.8% [−17.8%: −14.9%], *p* = 0.072), but did with the peak-frame approach (SEG: −15.2% [−16.9%: −13.8%] vs. CS: −16.4% [−17.3%: −14.5%], *p* = 0.008). In contrast, the assessment of SCS by DRA using segment-based peaks was significantly different between acquisitions (SEG: −16.1% [−19.2%: −12.4%] vs. CS: −16.7% [−19.5%: −13.1%], *p* = 0.0059). Segmental strain generally showed wider interquartile ranges compared to global values.

**Table 3:** Comparison of global and segmental strain peak values (in %) between standard high-resolution acquisitions (SEG) and low-resolution compressed sensing acquisitions (CS)

Parameter	Software	Peak Type	SEG datasets	CS datasets	*P*-value
Global Circumferential Strain	FT	Frame	−17.9% [−19.7 : −16.8]	−15.9% [−17.0 : −13.0]	< 0.0001*
		Segment	−19.4% [−20.7 : −17.8]	−16.3% [−17.7 : −13.3]	< 0.0001*
	DRA	Frame	−15.2% [−16.9 : −13.8]	−16.4% [−17.3 : −14.5]	0.008*
		Segment	−16.0% [−17.5 : −14.4]	−16.8% [−17.8 : −14.9]	0.072
Global Radial Strain	FT	Frame	32.8% [28.9 : 37.2]	24.8% [18.4 : 27.1]	< 0.0001*
		Segment	35.1% [31.7 : 40.2]	25.5% [19.2 : 28.7]	< 0.0001*
	DRA	Frame	41.3% [35.4 : 45.2]	27.7% [23.8 : 29.7]	< 0.0001*
		Segment	43.2% [36.8 : 47.4]	28.4% [24.9 : 30.9]	< 0.0001*
Segmental Circumferential Strain	FT	Frame	−17.9% [−21.5 : −14.3]	−15.2% [−18.1 : −12.1]	< 0.0001*
		Segment	−19.0% [−22.4 : −15.6]	−15.6% [−18.4 : −12.7]	< 0.0001*
	DRA	Frame	−15.5% [−18.8 : −11.7]	−16.1% [−19.2 : −12.5]	0.0001*
		Segment	−16.1% [−19.2 : −12.4]	−16.7% [−19.5 : −13.1]	0.0059*
Segmental Radial Strain	FT	Frame	29.9% [21.4 : 40.7]	22.2% [16.3 : 28.8]	< 0.0001*
		Segment	33.0% [24.8 : 43.5]	23.1% [17.4 : 29.6]	< 0.0001*
	DRA	Frame	40.7% [26.9 : 51.8]	25.5% [13.7 : 38.3]	< 0.0001*
		Segment	42.4% [28.0 : 53.8]	26.3% [14.6 : 39.3]	< 0.0001*

FT and DRA stand for CVI 42 and Trufistrain, respectively. Values are presented as median [IQR] and compared using the Wilcoxon signed-rank test. A p-value of 0.05 was considered statistically significant, denoted by an asterisk (*)

### Agreement and equivalence testing

**Fig. 2:**
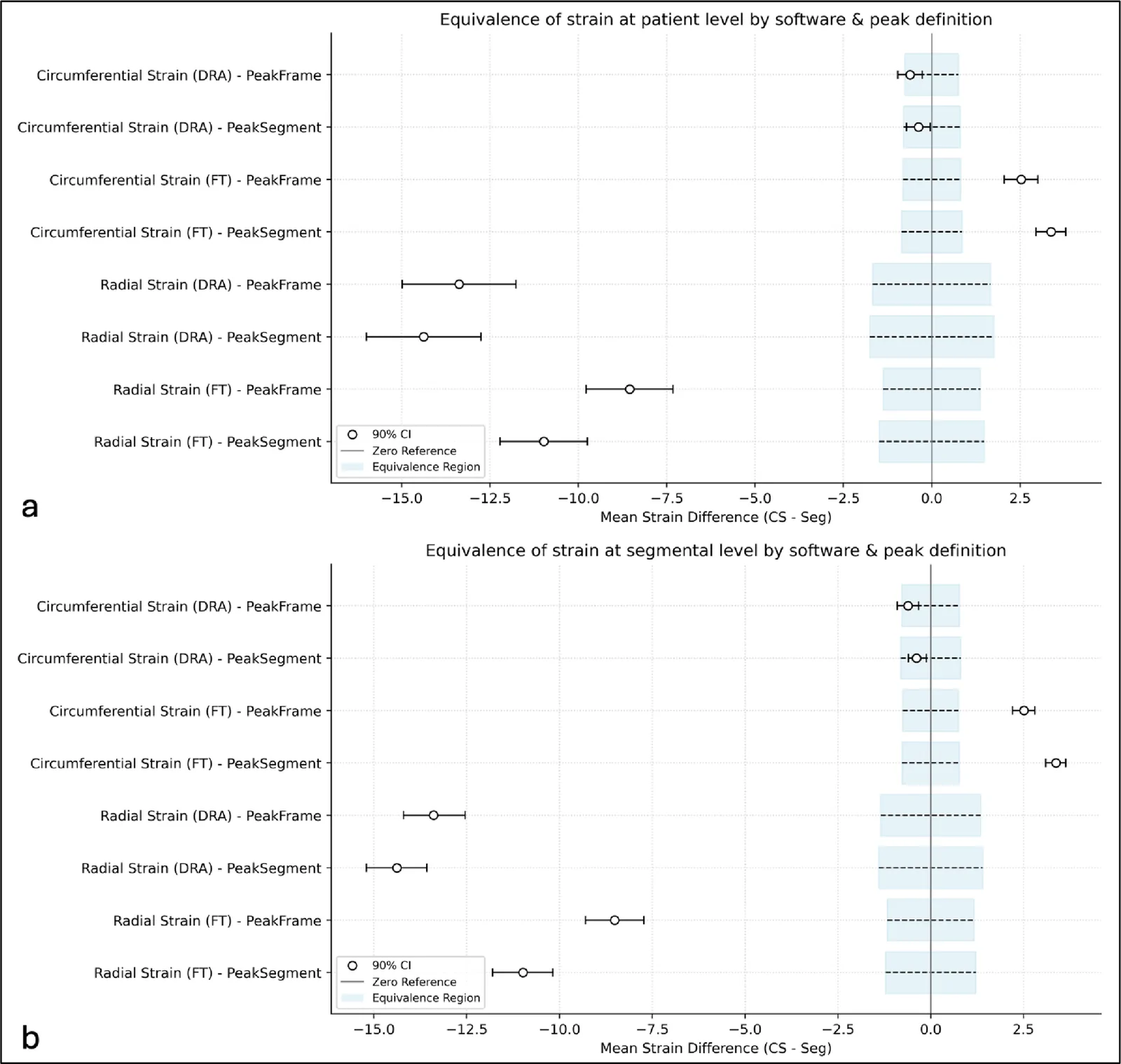
Equivalence analysis of global (a) and segmental (b) strain measurements between cine acquisition techniques and quantification methods. The equivalence regions (light blue) are derived from a ± 5% relative margin (calculated from the mean strain analysed across SEG and CS). Observed mean differences with 90% confidence intervals (horizontal bars) are shown for each quantification technique. Only one comparison (Circumferential Strain by DRA, using peak-segment methodology) achieved statistical equivalence at both the patient and segmental level, as its confidence interval lies entirely within the equivalence bounds

Equivalence of GCS, GRS, SCS, or SRS between SEG and CS acquisitions was assessed using TOST with 90% confidence intervals (CI90), using predefined equivalence bounds of ± 5% of mean peak strain values, Table 4; Fig. 2. Most comparisons did not meet the criteria for statistical equivalence, indicating non-equivalence between SEG and CS cines for these parameters at the 5% cut-off. Radial strain (global or segmental) showed the highest differences in peak values between SEG and CS.

Circumferential strain measured by DRA (segment-based peaks) demonstrated statistical equivalence between acquisitions at 5%, at both global (mean difference − 0.38%; CI90 [−0.72% : −0.05%]; bounds ± 0.80) and segmental levels (mean difference − 0.38%; CI90 [−0.64% : −0.12%]; bounds ± 0.81). The equivalence findings were robustly powered (0.977 global, 0.999 segmental).

**Table 4:** Equivalence analysis of global and segmental strain peak values

Parameter	Software	Peak type	Mean difference (CI90)	Equivalence bounds	Equivalence	TOST power
Global Circumferential Strain	FT	Frame	2.53% [2.05 : 3.00]	[−0.81, 0.81]	No	0.766
		Segment	3.38% [2.95 : 3.80]	[−0.86, 0.86]	No	0.915
	DRA	Frame	−0.62% [−0.97 : −0.27]	[−0.76, 0.76]	No	0.955
		Segment	−0.38% [−0.72 : −0.05]	[−0.80, 0.80]	Yes	0.977
Global Radial Strain	FT	Frame	−8.55% [−9.78 : −7.32]	[−1.38, 1.38]	No	0.183
		Segment	−10.98% [−12.21 : −9.75]	[−1.49, 1.49]	No	0.275
	DRA	Frame	−13.37% [−14.99 : −11.76]	[−1.67, 1.67]	No	0.100
		Segment	−14.38% [−16.00 : −12.76]	[−1.76, 1.76]	No	0.275
Segmental Circumferential Strain	FT	Frame	2.51% [2.22 : 2.80]	[−0.75, 0.75]	No	0.991
		Segment	3.38% [3.11 : 3.64]	[−0.77, 0.77]	No	0.999
	DRA	Frame	−0.61% [−0.90 : −0.32]	[−0.78, 0.78]	No	0.995
		Segment	−0.38% [−0.64 : −0.12]	[−0.81, 0.81]	Yes	0.999
Segmental Radial Strain	FT	Frame	−8.51% [−9.30 : −7.73]	[−1.16, 1.16]	No	0.578
		Segment	−10.98% [−11.78 : −10.17]	[−1.21, 1.21]	No	0.600
	DRA	Frame	−13.39% [−14.23 : −12.54]	[−1.35, 1.35]	No	0.680
		Segment	−14.38% [−15.19 : −13.57]	[−1.40, 1.40]	No	0.780

Equivalence testing of global and segmental strain peak values (in %) between standard high-resolution acquisitions (SEG) and low-resolution compressed sensing acquisitions (CS). FT and DRA stand for CVI 42 and Trufistrain, respectively. Mean differences between peak strain results are reported with 90% confidence intervals (CI90). Equivalence bounds, set at 5% of the mean peak strain values, define the range within which SEG and CS are considered statistically equivalent. Equivalence is established when the CI90 of the mean difference falls within the 5% equivalence bounds. Post-hoc power analysis results of the two one-sided test (TOST) procedures are included

**Fig. 3:**
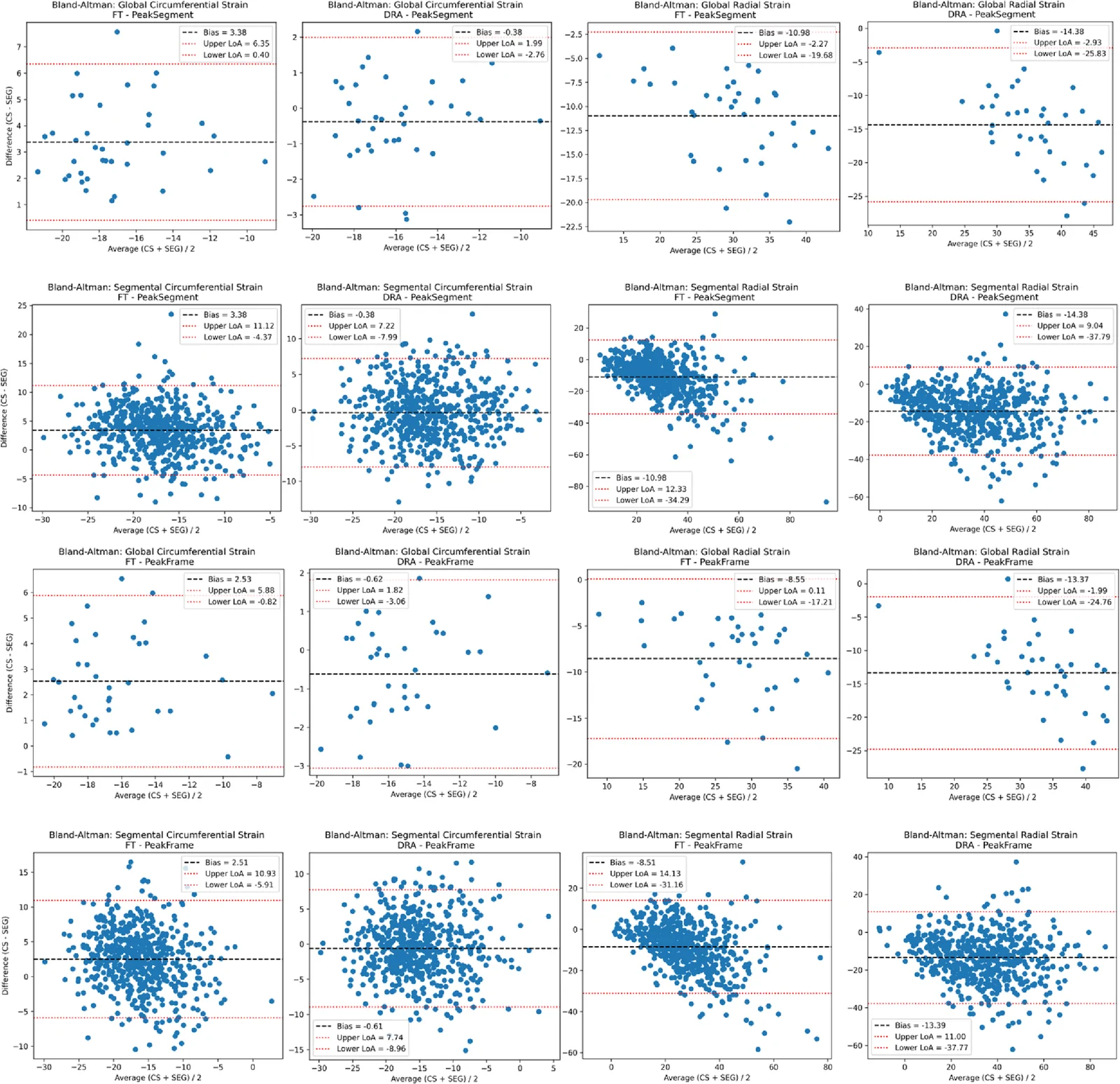
Bland–Altman plots showing agreement between sequences, using both peak–segment strain measurements (top two rows) and peak–frame strain measurements (bottom two rows). Each pair of rows shows agreement in global strain in the first row and segmental strain in the second. Dashed horizontal lines indicate the mean bias (black) and the 95% limits of agreement (red). Most strain values are within the limits of agreement. However, notice the wider limits of agreement for radial strain across both software and the significantly larger bias in circumferential strain for the FT software

Bland–Altman analyses showed interchangeable trends between frame- and segment-based peak approaches (Fig. 3). Generally, GCS across sequences showed a wider limit of agreement with FT (bias: 3.38%; LoA: 0.40% – 6.35%) than with DRA (bias: −0.38%; LoA: −2.76% – 1.99%), which was consistent with equivalence testing. SCS across sequences had wider limits of agreement than GCS but remained comparable between FT (bias: 3.38%; LoA: −4.37% – 11.12%) and DRA (bias: −0.38%; LoA: −7.99% – 7.22%). On the other hand, GRS and SRS consistently demonstrated larger inter-sequence differences and broader limits of agreement (bias ranging from − 10.98% to − 14.38%; LoA up to − 37.79% – 9.04%) across software, with slightly narrower limits observed for FT-derived measurements.

### Correlation analyses

**Fig. 4:**
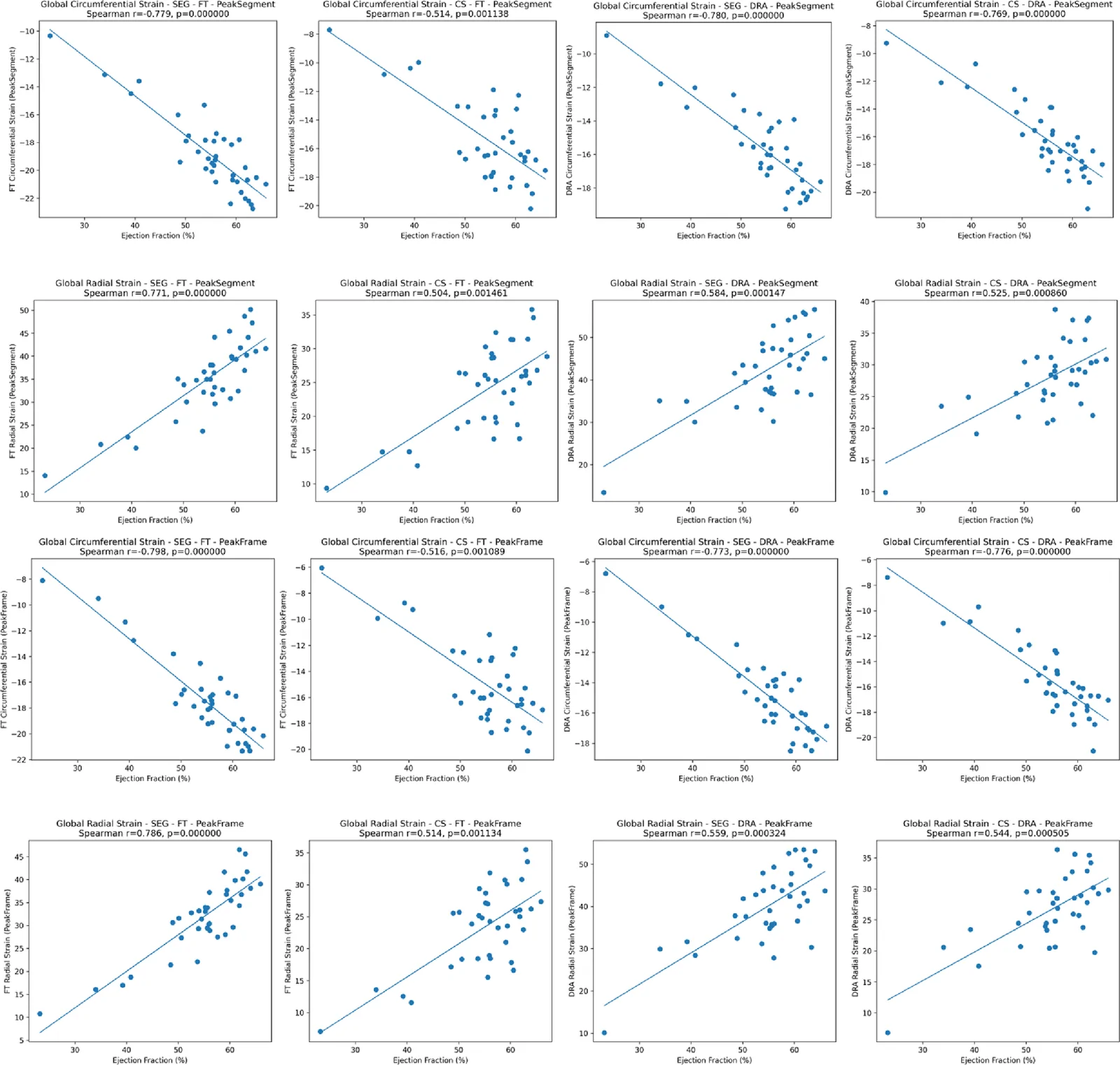
Spearman correlations between global strain and left ventricular ejection fraction (LVEF) across cine imaging techniques: segmented high-resolution (SEG) and compressed-sensing low-resolution (CS). Each row shows a combination of peak definition, sequence, and software for a specific strain (radial or circumferential). Scatter plots include fitted regression lines; Spearman’s ρ and corresponding p-values are displayed in each panel. All technique–software–strain combinations correlate significantly with LVEF. Notably, correlation coefficients decrease from SEG to CS images for FT, but not for DRA

GCS and GRS significantly correlated with LVEF across all combinations of sequence (SEG, CS) and software (FT, DRA), Fig. 4. FT-derived GCS from SEG images showed a stronger correlation with LVEF than from CS images (Spearman’s ρ = −0.78 vs. −0.51, respectively). In contrast, DRA correlation coefficients between GCS and LVEF were similar using either SEG or CS images (Spearman’s ρ −0.78 vs. −0.77, respectively). Radial strain generally showed lower correlations with LVEF, except FT-derived radial strain from SEG images, which correlated strongly (Spearman’s ρ = 0.77).

## Discussion

In this study, it was assessed whether reduced spatial resolution combined with compressed sensing (CS) acceleration affects myocardial strain measurements (circumferential and radial) derived from two different feature-tracking packages. Overall, both circumferential and radial strain at global and segmental levels differed significantly between high-resolution segmented images (SEG) and low-resolution single-shot images (CS). A single combination (circumferential strain using segment-based peaks) was found to be equivalent at a 5% cut-off across sequences, but only for one software.

Variability is the primary limitation of myocardial strain and has been widely documented in the literature [7]. Inter-vendor heterogeneity is consistently identified as the most significant contributor to this variability, whereas differences between observers and scanners are considered minor, especially with the widespread adoption of automatic contouring schemes [12]. The present study demonstrates that sequence-related factors, specifically reduced spatial resolution and CS acceleration, also introduce meaningful differences within a single software package. Because meta-analyses reporting reference values may not explicitly report the cine sequence used [9], awareness of inter-sequence variability must be improved. In practice, cardiac imagers should carefully consider the type of cine sequence used when interpreting or publishing strain data, particularly in heterogeneous patient cohorts in which free-breathing or single-shot acquisitions are required.

As spatial resolution decreases and voxel size increases, strain measurements by feature-tracking have been shown to be underestimated relative to ground-truth deformation, consistent with the hypothesis that displacement information is lost within voxels [19]. In contrast, reports on the impact of CS acceleration alone have shown mixed results: Kido et al. observed that GCS was not significantly different, with even a tendency of slightly higher values [21], whereas Halfmann et al. reported significantly lower GCS using the same two software packages as in this study [22]. We report results from a different, combined approach of reduced spatial resolution and compressed sensing. Radial strain was markedly underestimated using CS datasets (≈ 50% lower than high-resolution data), while circumferential strain showed software-dependent behavior: either slightly overestimated (≈ 15% higher than high-resolution data) or close to equivalence.

Notably, circumferential strain derived from one software using the peak-segment approach demonstrated statistical equivalence between SEG and CS datasets at both global and segmental levels. Prior work has emphasized the reproducibility of this software for GCS assessment in healthy volunteers and hypertrophic cardiomyopathy [23, 24], but robustness to image degradation has not previously been described. Although many algorithmic differences exist between the evaluated packages, one key distinction may contribute to this finding: the software showing equivalence performs full-wall tracking, estimating motion from pixel-wise displacement across the entire myocardial thickness, whereas the other tracks features along the endocardial and epicardial contours. As a result, in either high- or low-resolution image conditions, the number of features tracked was higher. The improved consistency observed with full-wall tracking under reduced resolution may therefore reflect a statistical effect, in which tracking more features (i.e., pixels) reduces measurement variance, analogously to increasing the sample size of an estimator, and is consistent with prior work in computer vision and medical image registration [29, 30]. Additional proprietary differences between software packages likely contribute to their differential sensitivity to sequence changes, but these differences are generally inaccessible to clinical end-users. Therefore, each package should be evaluated across the sequences used in clinical practice, as algorithms may respond differently to variations in image resolution.

Strain equivalence between SEG and CS cine sequences was achieved only when strain was derived using the peak-segment approach. In contrast, peak-frame values did not meet equivalence criteria, although they approached significance. Prior studies comparing cine sequences have not used peak-segment methods, which may explain discrepancies with our results. Clinically, most vendors report peak-frame strain, but segmental values by frame are typically accessible in the software backend and can be processed for research. The peak-segment approach is more frequently used in echocardiography [31] to evaluate mechanical dispersion and, to a lesser extent, in MRI [32]. Using it more systematically may allow more precise characterization of true segmental peak contraction and could represent a promising avenue for improving reproducibility in strain imaging.

This study has some limitations. First, we did not compare strain values with reference techniques such as myocardial tagging or DENSE. Absolute strain accuracy for either sequence or software could therefore not be assessed, even if strain differences are known [5, 9]. Second, a relatively modest sample size needs to be acknowledged. Although the study was sufficiently powered to detect both significant differences and equivalence between paired measurements, generalizability remains limited, especially to patients with limited breath-hold capacity or those unable to tolerate conventional imaging. Third, longitudinal strain analysis was not performed because paired long-axis cine images were unavailable in some patients. Fourth, reproducibility was not assessed, as contouring was automated and comparability was ensured through standardized quality control. Fifth, interpolation of CS cines has been shown to reduce strain measurements compared to non-interpolated images [33]. However, given the high number of native frames in our dataset, such effects are expected to be minimal. Finally, the reported findings apply specifically to the software versions, scanner platform, and cine sequence variants tested in this study.

In conclusion, cine image degradation resulting from reduced spatial resolution and compressed sensing acceleration significantly affects myocardial strain measurements. Cross-sequence equivalence cannot, therefore, be assumed, even when using a single software platform and its specific strain ranges [10]. Radial strain was particularly sensitive to image changes, whereas circumferential strain showed variable robustness across software and peak-definition methods. Only one of eight tested combinations of strain parameter, software, and peak-definition demonstrated statistical equivalence between SEG and CS images. These findings highlight the complex interplay between imaging protocol, post-processing software, and peak definition, emphasizing the need to consider all three factors to ensure reproducible and clinically reliable strain evaluation.

## Data Availability

No datasets were generated or analysed during the current study.

## Abbreviations

CMR: Cardiac Magnetic Resonance
CS: Compressed Sensing
DRA: Deformable Registration Analysis
FT: Feature-tracking
GCS: Global Circumferential Strain
GRS: Global Radial Strain
LVEF: Left Ventricle Ejection Fraction
MRI: Magnetic Resonance Imaging
PACS: Picture Archiving and Communication System
SCS: Segmental Circumferential Strain
SRS: Segmental Radial Strain
SSFP: Steady-state Free Precession
